# New Insights into Neuromuscular Junction Biology: Evidence from Human and Animal Research

**DOI:** 10.3390/ijms27031253

**Published:** 2026-01-27

**Authors:** Zhanyang Liang, Xiaoying Chen, Mahtab Nourbakhsh

**Affiliations:** Institute of Pathology, RWTH Aachen University Hospital, 52074 Aachen, Germany; zliang@ukaachen.de (Z.L.); xchen@ukaachen.de (X.C.)

**Keywords:** neuromuscular junction, remodeling, regeneration, transduction, acetylcholine receptor, synaptic vesicle protein

## Abstract

Neuromuscular junctions (NMJs) are highly specialized synapses that enable efficient communication between motor neurons and skeletal muscle fibers. Impaired formation or maintenance of NMJs is implicated in the pathogenesis of multiple neuromuscular disorders and contributes to age-related declines in skeletal muscle mass and strength. NMJ functionality is governed by complex regulatory crosstalk among different cells and is mediated by a diverse network of proteins. Moreover, immune cells often reside at NMJs and exhibit phenotypically different characteristics depending on the regenerative state of the muscle. These complex interfaces have posed a significant challenge for elucidating pathogenic mechanisms and developing biomarkers or effective targeted treatments. Many animal models have been developed to address this challenge by characterizing the fundamental structural features of neuromuscular junctions (NMJs) and their transmission capacity under both healthy and disease conditions. In contrast, studies of human NMJs remain limited, although emerging evidence is increasingly revealing substantial morphological and functional differences from animal NMJs. This review provides an overview of animal research on NMJs over the past decades, highlighting interspecies differences and key advances in our understanding of human NMJs.

## 1. Introduction

Neuromuscular junctions (NMJs) are specialized cholinergic junctions in mammals that mediate skeletal muscle contraction through peripheral synapses. Situated at the terminus of motor axons, they enable rapid and reliable transmission of neural signals to drive both voluntary movements and involuntary reflexes. Therefore, the structural integrity and functional stability of NMJs are crucial for maintaining motor control and overall neuromuscular function.

Building on early descriptions of the “motor endplate” in frog muscles by Wilhelm Friedrich Kühne in 1862 [1], and foundational principles of synaptic physiology originating from invertebrate models by Bernard Katz’s pioneering work [2], NMJs have been examined in rodents, particularly in mice, since the 1970s [3]. Degenerative or dysfunctional changes in this microstructure are closely associated with multiple neuromuscular disorders and with age-related motor decline. Accumulating evidence from animal models indicates that the morphology and transmission efficiency of NMJs are not predetermined; instead, NMJs undergo continuous remodeling under diverse physiological and pathological contexts, including development, aging, autoimmunity, trauma, metabolic imbalance, and neurodegenerative conditions [4,5]. This plasticity highlights NMJs as dynamic and sensitive signaling hubs whose status may reflect the overall integrity of the neuromuscular system.

Recent animal studies have increasingly focused on the mechanisms that govern NMJ remodeling and regeneration, especially how nerve, muscle, and immune components interact to maintain synaptic stability or promote postdamage repair [6,7,8]. Understanding these intercellular and mechanical transduction processes in human NMJs [9,10] may provide a conceptual framework for the development of interventions aimed at maintaining or restoring neuromuscular connections in states of health, aging, and neuromuscular disorders such as myasthenia gravis (MG) [11], spinal muscular atrophy (SMA) [12], and amyotrophic lateral sclerosis (ALS) [13].

## 2. NMJ Structure and Regulatory Components

**Figure 1:**
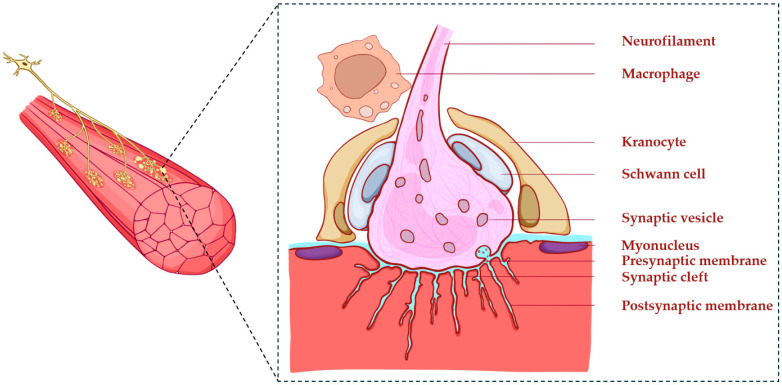
Schematic representation of NMJ organization. Motor neurons extend toward the surface of muscle fibers and form clusters of terminal branches (**upper left**). Each NMJ consists of the presynaptic terminal of the motor neuron, the synaptic cleft, and the postsynaptic membrane of the muscle fiber associated with a myonucleus. Schwann cells, kranocytes, and macrophages are commonly found in close proximity to NMJs.

NMJs consist of three main domains: the presynaptic terminal of the motor neuron, the synaptic cleft, and the postsynaptic membrane of the muscle fiber (Figure 1) [14]. Additionally, NMJs are surrounded by different types of cells that possess diverse regulatory functions, including maintenance of the synaptic structure, early damage sensing, and regulation of inflammation. The best featured NMJ-associated cells are Schwann cells, named after the German physiologist Theodor Schwann. They play a crucial role in the development and maintenance of NMJs [6]. Other cells, including kranocytes and immune cells, are commonly found near NMJs [7,15]. However, their specific contributions to NMJ development and repair have yet to be systematically investigated. Other components, such as mast cells [16] and specialized myonuclei [17], also contribute to this complex regulatory environment.

### 2.1. Presynaptic Terminals

**Figure 2:**
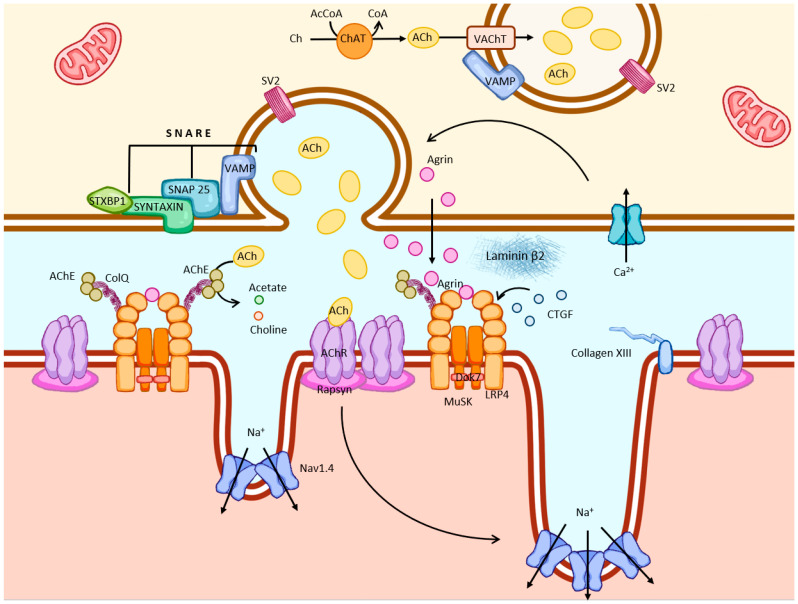
Schematic presentation of neurotransmitter effects and signaling mechanisms within NMJ pre- and postsynaptic membranes.

Presynaptic motor endings mediate the synthesis, storage, and secretion of the neurotransmitter acetylcholine (ACh) (Figure 2). Early studies of NMJs in frog, zebra finch, and Chinese hamster have established that motor axons arise from neurons in the spinal cord and project toward skeletal muscle fibers, where they branch into terminal axonal endings that form specialized synaptic structures known as motor endplates [1]. Each synaptic body appears as a small spherical enlargement at the motor nerve terminal and is densely filled with synaptic vesicles that store ACh. In nerve endings, ACh is synthesized by choline acetyltransferase (ChAT), which drives the production of ACh from choline and acetyl coenzyme A.

Across vertebrate species, terminal axonal endings exhibit characteristic patterns of arborization. As motor axons approach the muscle surface, they disperse into multiple terminal branches that culminate in bouton-like swellings enriched in synaptic vesicles. This branching pattern establishes the core presynaptic architecture of the NMJ and determines the spatial distribution of neurotransmitter release sites along the postsynaptic membrane. Notably, compared with human NMJs, rodent NMJs are more complex and have more extensively branched terminal arbors. Human presynaptic terminals exhibit markedly simplified morphology with fewer branch points—reflecting a species-specific reduction in arbor complexity rather than diminished synaptic capacity [18,19].

In addition to spatial patterns, studies of mouse embryos revealed that presynaptic terminal composition is governed by molecular interactions, involving the binding of low-density lipoprotein receptor-related protein 4 (LRP4) to the CT domain of connective tissue growth factor (CTGF/CCN2) [20]. Loss-of-function analyses revealed that the connective tissue growth factor CTGF (CCN2) supports LRP4–MuSK signaling to maintain proper synaptic vesicle clustering within terminal boutons. In CTGF knockout (KO) mice, synaptic vesicles are distributed aberrantly along motor axons instead of being restricted to presynaptic endings [20]. These findings indicate that terminal arborization and bouton maturation depend on coordinated extracellular regulatory cues during development [20].

Murine presynaptic terminals have been extensively studied, leading to the identification of a variety of key proteins that regulate vesicle circulation and neurotransmitter release, including the core elements of the soluble N-ethylmaleimide-sensitive factor attachment protein receptor (SNARE) complex, the synaptic binding protein, and the synaptic vesicle protein 2 (SV2) [21]. These molecular mechanisms work together to mediate the biosynthesis, membrane fusion, and recycling of vesicle contents. Previous investigations in transgenic mouse models revealed that action potentials at nerve endings, followed by Ca^2+^ influx, initiate the fusion of neurotransmitter-filled synaptic vesicles, thereby facilitating rapid signal transmission across the synaptic cleft [21]. Furthermore, recent studies in mouse and cell culture models have indicated that additional proteins, including RIM, Munc13, and ELKS, form a dynamic molecular scaffold that couples Ca^2+^ influx with synaptic vesicle fusion, thereby regulating neurotransmitter release with nanometer-scale precision [22,23,24].

Recent high-speed imaging of single-channel Ca^2+^ nanodomains of the large reticulospinal presynaptic terminals in lamprey revealed that Ca^2+^ entry at individual active zones may be mediated by only a few—sometimes even a single—voltage-gated calcium channel [25]. Such single-channel nanodomains enable exceptionally tight spatial coupling between Ca^2+^ influx and synaptic vesicle fusion, thereby supporting the fast (1–4 ms) release kinetic characteristics of vertebrate NMJs [25].

Other studies in mouse and frog models have reported finely tuned mechanisms maintaining cholinergic homeostasis at the NMJ. In particular, acetylcholinesterase (AChE) is precisely distributed within the synaptic cleft, reflecting a tightly regulated microenvironment for ACh turnover [26]. These findings suggest that cholinergic synthesis activity may be dynamically regulated in response to exercise demands and neuronal activity.

### 2.2. Synaptic Cleft and Basal Lamina Structures

The synaptic gap is a narrow extracellular space that separates the presynaptic end from the postsynaptic muscle membrane. The synaptic cleft at the human NMJ was first described in ultrastructural detail by Murata and Ogata (1969), who demonstrated in the intercostal muscle that the narrow space between the presynaptic terminal and the tops of the junctional folds is filled with dense, basal-lamina-like material [14]. Comparative studies using both human primary myotubes and murine muscle cells have confirmed that laminin β2 and agrin cooperatively activate the MuSK–Lrp4 signaling axis to promote focal clustering of acetylcholine receptors (AChRs) at the postsynaptic membrane [27]. Consistently, human muscle biopsies from congenital myasthenic patients carrying AGRN or LRP4 mutations show disrupted AChR organization and fragmented basal lamina, which are phenotypes faithfully recapitulated in animal models, underscoring the conserved role of agrin–laminin–MuSK–Lrp4 signaling in NMJ development and long-term stability [28]. In cell culture experiments, agrin was capable of enhancing the internalization of MuSK and its downstream signals independently [29]. These findings suggest the involvement of a secondary regulatory pathway that can alter receptor turnover and postsynaptic membrane tissue [29].

The structural component of the basal membrane, collagen XIII, was implicated in NMJ repair in collagen XIII-deficient C57BL mice [30]. During NMJ formation, collagen XIII is crucial for not only the organization of the synaptic region but also the formation and regeneration of axons during the repair process [30]. Evidence from human muscle biopsies indicates that AChE—another key constituent of the synaptic basement membrane—is precisely localized along the synaptic cleft and postsynaptic folds, which is consistent with the conserved ultrastructural organization of the human NMJ [31]. This was assumed to ensure full hydrolysis of ACh by AChE, thus eliminating its reattachment to its receptor. Building upon earlier genomic studies that linked COLQ mutations to endplate AChE deficiency in humans [32,33], recent investigations using patient-derived muscle biopsies and iPSC models confirmed that C-terminal COLQ variants impair COLQ–LRP4 interactions, leading to defective AChE anchoring and abnormal synaptic organization at the human NMJ [34].

### 2.3. Postsynaptic Structural–Functional Domain

The postsynaptic membrane is a deeply in-folded specialized part of muscle fibers. Its main feature is extensive connections and folding, which significantly increase the area of receptor distribution and improve synaptic transmission efficiency. Studies in human tissue and cells have demonstrated that this site contains high concentrations of nicotinic AChRs (nAChRs) and various key regulatory proteins, including MuSK, LRP4, Dok-7, and Rapsyn [35]. These components work together to coordinate signal transduction related to aggregation, anchoring, and downstream receptors, helping to maintain the architecture and functional integrity of the NMJ.

ACh combines with nAChRs, leading to channel opening of non-selective cation, allowing Na^+^ influx and K+ efflux, generating an endplate potential. When the EPP reaches the threshold, Nav1.4 channels open, and the resulting action potential travels along the sarcolemma to initiate contraction, resulting in the translation of synaptic input into force [36,37]. Continuous structural research has improved this mechanism. Recent cryo-electron microscopy (cryo-EM) studies of human and mammalian nAChR have provided near-atomic insights into receptor architecture. Cryo-electron microscopy (cro-em) is capable of analyzing the substructures of nAChR with near-atomic precision and visualizing the conformational transitions during receptor activation and desensitization processes [38,39]. These structural findings clarify how chemical transmission is converted into electrical excitation at the NMJ and provide a basis for the structure-guided development of targeted modulators applicable to disorders such as myasthenia gravis.

Morphologically, motor nerve endings are embedded in the primary synaptic sulcus on the muscle surface, while the postsynaptic membrane protrudes inward to form secondary folds. These infoldings increase the size of the receptive surface and improve the efficiency of synaptic transmission. The acetylcholine receptor (AChR) is enriched on the crests of the junctional folds and positioned opposite presynaptic active zones, as confirmed in the human NMJ using expansion microscopy [40]. In contrast, Nav1.4 channels are concentrated deep within the folds in the mouse NMJ, facilitating local depolarization and reliable action-potential propagation [41]. Despite the existence of this clear spatial separation, the molecular mechanisms that determine the depth and periodicity of these folds remain unexplained. Recent evidence suggests that the AChR γ-to-ϵ subunit switch is critical for NMJ maturation, as incorporation of the ϵ-subunit promotes the stabilization and deepening of junctional folds, thereby establishing proper endplate morphology and periodicity [38,40].

Rodent research has shown that the formation of folds continues after birth, which may be related to the insertion of membranes rich in Nav1.4, which drive folds to expand inward [42]. In addition, super-resolution imaging has revealed that caveolin-3-dependent membrane infolding can occur even in the absence of nerve input, indicating a nerve-independent, lipid-raft driven mechanism for structural maturation [43]. Although these models are not mutually repulsive, they jointly emphasize the high regulation and dynamism of the postsynaptic membrane structure. During regeneration and repair, re-expression of the fetal γ-subunit transiently restores synaptic responsiveness and facilitates structural remodeling of the postsynaptic folds, suggesting that the subunit composition dynamically modulates NMJ recovery [37,44].

### 2.4. Terminal Schwann Cells: Key Regulators of NMJ Homeostasis and Repair

Schwann cells are non-myelinating glial cells that mainly wrap around the outer surface of the presynaptic terminal but also extend small protrusions around the synaptic gap, and some protrusions even reach the edge of the synaptic space [6,45]. Notably, presynaptic vesicles co-release ATP alongside acetylcholine; terminal Schwann cells (tSCs) sense this purinergic signal to trigger calcium-dependent signaling, thereby establishing the NMJ as a functional “tripartite synapse” where glial cells actively monitor and modulate synaptic activity [46,47]. These presynaptic Schwann cells (PSCs) play an indispensable regulatory role in the assembly, maturation, maintenance and regeneration of NMJs. Human histopathological studies corroborate these findings: in muscle biopsy samples from patients with amyotrophic lateral sclerosis, PSCs (presynaptic Schwann cells, also termed tSCs) exhibit abnormal cytoplasmic projections extending into the synaptic cleft, whereas in congenital myasthenic syndromes caused by LAMB2 or AGRIN mutations, tSCs are either absent or structurally disorganized [48,49].

### 2.5. Kranocytes: Structural Positioning and Functional Hypothesis

Court et al. [15] first systematically identified a group of extralayer cells above the NMJ, later known as synaptic kranocytes. Mouse and rat models were used for comparison with amphibian and avian NMJs. These cells are present in the junction area during the early development process and exhibit strong reactivity, proliferative ability, and migratory behavior after damage.

Experimental evidence suggests that synaptic nuclear cells may play a role in synaptic remodeling following nerve damage and are even activated before terminal Schwann cells initiate axon regeneration. These findings suggest that synaptic nuclear cells may play a role as early responders and regulators in the maintenance and repair of NMJs [15].

### 2.6. NMJ-Associated Myonuclei

In addition to the classic three-part structure of the NMJ—comprising the presynaptic terminal, synaptic space, and postsynaptic membrane—recent studies have identified a group of functionally specialized muscle nuclei located in the muscle fibers near the NMJ (approximately 10–40 µm in mice), called NMJ auxiliary nuclei [50].

In a recent single-cell and mononuclear transcriptomics study combining spatial positioning, researchers constructed a comprehensive spectrum of human skeletal muscle aging and identified several “specialized nuclear clusters”, including NMJ auxiliary nuclei. The expansion of the distribution of these nuclei in the muscles of elderly individuals may reflect the compensatory redomination response to age-related denervation [17]. In contrast, during disuse atrophy, such as bed rest, myonuclear domains shrink, while subsynaptic myonuclei are preferentially preserved, maintaining NMJ integrity and enabling faster recovery upon reactivation [51,52].

### 2.7. NMJ-Associated Immune Cells

Previous studies reported the increased accumulation of mast cells and macrophages near degenerating motor axons and NMJs in a amyotrophic lateral sclerosis (ALS) rat model [37]. Furthermore, the accumulation of mast cells and macrophages was successfully prevented by systemic treatment with masitinib, a tyrosine kinase inhibitor that has shown beneficial effects in clinical trials for ALS [37]. In rodents, peripheral nerve injury induces immune activation within muscle, including macrophage accumulation near denervated endplates [7]. These findings provided evidence for the implication of mast cells and macrophages in distal axonopathy. Consistent with these findings, nerve injuries led to macrophage accumulation close to NMJs in an ALS mouse model [16,53]. In another ALS mouse model, sustained immune activation around degenerating NMJs was associated with impaired regeneration, while immune modulation partially preserves NMJ structure [54]. Mechanistically, macrophages facilitate the clearance of synaptic debris and modulate the inflammatory milieu that supports axonal regrowth and endplate remodeling [8]. Although these observations propose immune cell infiltration and NMJ instability as potential pathogenic defects in rodents, their role in the human system is unknown. Studies of human skeletal muscle established the implication of tissue-resident macrophage populations in aging, chronic inflammation, and tissue regeneration [55,56,57]. Although NMJ instability and immune dysregulation frequently co-occur in human aging and neuromuscular diseases, the functional relevance of perisynaptic macrophages in humans remains to be established.

### 2.8. Interspecies Differences

Recent studies using high-resolution imaging and proteomics have reported significant structural differences between NMJs in humans and those in other species. Among the NMJs of all studied mammalian species, human NMJs are the smallest and have the lowest quantity of ACh discharged by a nerve impulse, which is less than 50% that of mice [18]. Furthermore, although the terminal plate area of human NMJs is considerably smaller despite the greater diameter of human muscle fibers, this limitation is likely compensated by deeper and more intricate junctional infoldings [19]. These morphological specializations substantially expand the postsynaptic surface area—by approximately eightfold—thereby increasing receptor density and enhancing synaptic transmission efficiency.

Detailed morphological analysis revealed that the human NMJ shows a higher degree of fragmentation, longer presynaptic axons, and simpler neural endings, and its terminal plate has a discrete “coin-shaped” structure [18]. Extensive folding of the postsynaptic membrane significantly increases the density of the AChR, thus optimizing the synaptic performance in a compact structure [40].

Boehm et al. conducted a comparative analysis of six mammals, including humans, human-like primates, and standard experimental animals, and the results revealed significant interspecific heterogeneity in the volume and morphology of the NMJ [19]. In this study, the average area of the human NMJ terminal plate was approximately 194 μm^2^, and the postsynaptic membrane area was 99 μm^2^, both of which are significantly smaller than those of mice (387 μm^2^ and 237 μm^2^ respectively), although the diameter of human muscle fibers was larger (53 μm vs. 30 μm) [19].

Notably, the NMJ of mice is the largest and most complex “pretzel-like” terminal form, whereas the NMJ of cats is the smallest. In contrast, the NMJs of sheep and pigs are similar and more closely related to the structure of human NMJs, indicating that large animal models may provide a more representative and transformative system for the study of the physiology and pathology of human NMJ.

Furthermore, comparative analyses revealed substantial morphological heterogeneity of NMJs across species and among different muscle groups within the same species. This variability is believed to mainly stem from the intrinsic characteristics of the motor neurons themselves rather than the differences in muscle fiber types. Table 1 provides a comparative overview of studies on NMJ structural and regulatory components across different experimental models, linking mechanistic targets to disease contexts and potential clinical translation.

## 3. NMJ Remodeling and Regeneration

### 3.1. NMJ Structural Remodeling and Adaptation

The neuromuscular junction (NMJ), once regarded as a rigid and structurally fixed synapse, is now understood to be a highly dynamic and plastic interface capable of extensive structural and functional remodeling. This intrinsic plasticity allows the NMJ to adjust its synaptic efficacy and architecture in response to developmental signals, changes in mechanical load, metabolic challenges, or pathological conditions. Through such remodeling, the NMJ preserves reliable motor output across a wide spectrum of physiological states.

In rodent models, NMJ properties undergo continuous adaptive modification in response to altered contractile demands [66]. Increased neuromuscular activity during sustained or high-intensity exercise promotes synaptic expansion and increases the density of active zones and the efficiency of neurotransmission [66]. Deepening of postsynaptic junctional folds represents a key structural adaptation closely linked to improved synaptic performance. Conversely, prolonged disuse or mechanical unloading induces presynaptic atrophy, reduced terminal arborization, and disruption of postsynaptic AChR organization, collectively diminishing synaptic efficacy and accelerating functional decline [65]. In humans, endurance training increases the stability and transmission efficiency of terminal-plate AChRs. Electrophysiological indices—including compound muscle action potential (CMAP) amplitude and tremor characteristics—also improve with training, demonstrating that the human NMJ exhibits measurable functional plasticity [60].

Furthermore, recent injury model studies have demonstrated that presynaptic and postsynaptic compartments display asymmetric structural susceptibility to toxic or metabolic stress [68]. In a toxin-induced NMJ injury model in adult mice, presynaptic nerve-terminal branching remained largely intact despite significant neuromuscular damage, whereas postsynaptic AChR domains exhibited marked reductions in endplate area, decreased perimeter length, and increased spatial fragmentation [68]. These observations indicate that, under pathological conditions, the presynaptic terminal often retains much of its architectural integrity, whereas the postsynaptic membrane undergoes predominant structural remodeling during both degenerative and reparative processes.

To date, multiple regulatory pathways have been implicated in the maintenance and regenerative capacity of NMJ:

- Multiscale regulatory networks

Evidence from human neuromuscular tissue and ex vivo reinnervation models indicates that ECM remodeling and metabolic signaling jointly shape the regenerative potential of the NMJ, supporting a multilayered model of synaptic regulatory control [64].

- Metabolic signaling

The AMPK–PGC-1α axis integrates metabolic cues to regulate synaptic gene expression and AChR clustering, with its activity tightly modulated by contractile load and metabolic stress [65,67].

- Mechanotransduction

YAP/TAZ–TEAD signaling, together with focal adhesion kinase (FAK), maintains endplate architecture, stimulates synaptic-nuclear transcription, and reinforces cytoskeletal anchoring in response to mechanical loading [17].

- Neurotrophic cascades

Neurotrophic factors converge on the PI3K–Akt and MAPK pathways to preserve axonal integrity, promote AChR clustering, and sustain synaptic activation, thereby supporting NMJ stabilization during injury or metabolic stress [71].

- ECM-dependent control

Assembly and maintenance of the NMJ ECM are governed primarily by the agrin–LRP4–MuSK pathway, which establishes a molecular scaffold linking presynaptic release sites with postsynaptic AChR clusters. Human agrin–LRP4–MuSK complexes form stable supramolecular structures that remain responsive to fluctuations in the metabolic state [62].

- Muscle membrane mechanosensation

Beyond canonical integrin–FAK and YAP/TAZ signaling, the muscle membrane exhibits intrinsic mechanosensory properties that can prime the postsynaptic apparatus. Dynamic exposure of phosphatidylserine (PS) at the muscle surface acts as a molecular switch activating the mechanosensitive channel PIEZO1, driving Ca^2+^ influx and promoting myotube morphogenesis and membrane maturation. This mechanism illustrates how biophysical membrane properties and mechanotransduction directly support the postsynaptic remodeling of AChR domains [73]. Subsequent studies using genetic mouse models established a central role for PIEZO1 in muscle regeneration and satellite-cell activation via Rho-dependent signaling [10].

Collectively, these regulatory mechanisms define an integrated structure–function–adaptive framework in skeletal muscle that operates independently of the classical nerve-regeneration program. Whereas acute NMJ repair re-establishes synaptic connectivity and neurotransmitter release, these long-acting regulatory networks maintain the stability, resilience, and functional adaptability of NMJs across aging, shifts in mechanical demand, and metabolic stress.

### 3.2. NMJ Repair and Reinnervation

Successful reinnervation of the NMJ can occur following peripheral nerve injury in animal models. Nevertheless, the loss of neural input rapidly induces muscle fiber atrophy, disintegration of postsynaptic specialization, and disruption of intramuscular axons, ultimately compromising the efficiency of excitation–contraction coupling [74]. In parallel, studies in humans have indicated that the expression of acetylcholine receptor (AChR) subunits decreases, the number of postsynaptic folds declines, and synaptic transmission weakens following denervation [52]. During the subsequent process of reinnervation, regenerating motor axons rely on the residual presynaptic architectural framework to navigate back to their original synaptic territory. At this stage, terminal Schwann cells (tSCs) play a pivotal role. They undergo injury-induced morphological remodeling, extend dynamic cytoplasmic processes, and form directional guidance structures that channel nascent axonal sprouts toward the motor endplate, thereby ensuring the precision of synaptic reestablishment [75,76]. Furthermore, recent investigations of human NMJs—including those collected from the peroneus brevis muscle during lower-limb amputations and complemented by rectus abdominis samples from abdominal surgeries—confirm that tSCs consistently cap the adult human NMJ and display species-specific morphological features relevant to synaptic stability and remodeling [63]. Once regenerating axon terminals re-establish contact with muscle fibers, the presynaptic membrane reconstructs its active zones to support synchronous acetylcholine release. Concurrently, AChRs recluster within the postsynaptic membrane, and junctional folds are gradually reformed, collectively restoring effective neuromuscular transmission.

Functional NMJ recovery requires not only the reconstitution of its anatomical architecture but also the coordinated maturation of its biomechanical and electrophysiological properties. During the early stages of reinnervation, the NMJ structure remains fragile, and its physiological function is incompletely developed. As motor activity increases, mechanotransduction pathways—including focal adhesion kinase (FAK), the transcriptional coactivators YAP/TAZ, and integrin-dependent signaling cascades—are progressively activated. These pathways reinforce cytoskeletal anchoring, refine synaptic organization, and ultimately increase the stability and reliability of neuromuscular transmission [9]. Notably, although these mechanotransductive pathways have been well characterized in animal models and in human skeletal muscle more broadly, their direct involvement in adult human NMJ has not yet been experimentally demonstrated.

Growing clinical and histological evidence indicates that the extent of motor functional recovery is closely linked to the structural and functional stability of the NMJ. Intraoperative analyses of human upper-limb muscle biopsies—including those of the biceps brachii, flexor carpi, and abductor pollicis muscles—have shown that the preservation of motor endplates strongly predicts superior postoperative outcomes following peripheral nerve transfer surgery [77]. Consistently, biopsy studies of the human vastus lateralis muscle have shown that molecular destabilization of the NMJ parallels reductions in muscle contractile performance during periods of disuse, whereas reactivation through resistance training restores NMJ stability and increases synaptic transmission capacity [44]. On the other hand, the coordinated regeneration of axons, terminal Schwann cells, and postsynaptic structures supports the orderly reconstruction of the synapse, enabling the NMJ to recover its transmission efficacy and fatigue resistance, thereby sustaining normal muscle contractile function [44].

## 4. NMJ Degeneration Related to Age and Disease

Aging drives progressive remodeling of the NMJ microarchitecture, characterized by endplate fragmentation, partial synaptic withdrawal, reduced AChR density, and mitochondrial dysfunction [61]. During the early stages of aging, a limited degree of steady-state plasticity persists, enabling sporadic reinnervation events that temporarily preserve synaptic function [61]. However, chronic inflammation and sustained metabolic stress can deplete this compensatory reserve [69]. In conditions such as diabetes and muscular dystrophies, the resulting imbalance frequently manifests as denervation, immune-cell infiltration, and ECM sclerosis [64]. Collectively, these observations suggest that NMJ degeneration constitutes a critical convergence point for systemic metabolic and inflammatory disturbances associated with aging.

Neuromuscular aging reflects a gradual, multifactorial decline in synaptic integrity that precedes overt muscle atrophy and functional impairment [61]. While rodent studies consistently report pronounced terminal-plate fragmentation, synaptic retraction, and impaired neurotransmission, emerging human data reveal a more nuanced trajectory in which functional instability may arise without overt structural disintegration [18,61]. This divergence underscores the need to interpret aging-related NMJ remodeling within the specific physiological, metabolic, and regenerative context of humans rather than relying solely on rodent-based paradigms.

### 4.1. Evidence from Human Research

Early morphological studies have produced conflicting conclusions regarding the age-related deterioration of human NMJs. Classic autopsy analyses of external intercostal muscles have revealed preserved NMJ numbers and total synaptic areas across aging. However, more nuanced alterations—such as localized disruption of postsynaptic junctional folds and increased Schwann-cell reactivity—suggest the presence of low-grade ultrastructural remodeling rather than wholesale synaptic loss [78].

A landmark study by Jones et al. analyzed 2860 NMJs from amputated human lower-limb muscles and revealed that, although human NMJs are smaller and more highly fragmented than those of rodents, their overall morphology remains largely stable throughout adulthood [18,70]. However, because the specimens were harvested from individuals with vascular pathology and the study lacked healthy young longitudinal controls, this stability likely reflects a postmaturation plateau rather than true lifelong homeostasis.

A recent large-scale clinical investigation by Sarto et al. reframed the understanding of human NMJ aging by integrating electrophysiological, biochemical, and histological assessments across healthy, presarcopenic, and sarcopenic individuals [61]. Their findings reveal several key shifts:

- Functional impairment precedes clinical sarcopenia

Elevated single-fiber EMG jitter and reduced motor unit estimates (iMUNE) are evident in older adults, even in the absence of overt sarcopenia, indicating early neuromuscular transmission instability [61].

- Synaptic matrix remodeling

Circulating serum levels of the C-terminal agrin fragment (CAF)—a biomarker of NMJ ECM degradation—increase progressively with aging, indicating the occurrence of ongoing synaptic-matrix remodeling [61].

- Compensatory remodeling

The upregulation of caveolin-3 (Cav3) and the presence of NCAM^+^ fibers indicate active denervation–reinnervation processes, suggesting an endogenous compensatory attempt to preserve neuromuscular connectivity [61].

- Concurrent neural stress

Increased plasma neurofilament light chain (NfL), a marker of axonal damage, correlates strongly with electrophysiologic markers of synaptic instability, highlighting the involvement of parallel neural stress [61].

Together, these findings indicate that human NMJ aging can be detected well before clinically significant muscle atrophy emerges and that this process reflects dynamic, multidimensional remodeling rather than a simple linear decline. Furthermore, they position the NMJ as a crucial marker axis and shift the research perspective on age-related muscle weakness from a muscle-centric to a synapse-centric classification.

This shift represents a significant departure from conventional research, which was largely muscle-centric and relied on muscle biopsies to identify late-stage fiber atrophy and protein metabolic imbalances [79]. While those earlier studies primarily documented the end-stage structural results of aging, the synaptic-centric perspective identifies functional NMJ instability as the primary driver that precedes overt sarcopenia. Clinically, this allows for the early measurement of neuromuscular aging through sensitive physiological tools—such as single-fiber EMG jitter and iMUNE—complemented by circulating biomarkers such as C-terminal agrin fragment (CAF) and neurofilament light chain (NfL) [59]. These applications facilitate early risk stratification and provide a measurable window for intervention years before the manifestation of clinical muscle wasting.

### 4.2. Dysregulation of Inflammation and Oxidative Stress

Aging is often characterized by increased expression of inflammatory factors and a chronic, low-grade inflammatory state independent of infection, which is a major contributor to the pathogenesis of sarcopenia—the age-related loss of skeletal muscle mass and strength. A recent study in mice revealed that chronic low-grade inflammation increases NMJ vulnerability by increasing the expression of interleukin-6 (IL-6) and tumor necrosis factor-alpha (TNF-α), which activate the transcription factor NF-κB signaling pathway, suppress AChR expression, and destabilize postsynaptic architecture [69]. Concurrently, in vitro studies have revealed that extracellular vesicles derived from human bone marrow mesenchymal stem cells possess anti-inflammatory and antioxidant properties in the mouse skeletal muscle myoblast cell line C2C12 [80]. Furthermore, oxidative stress impairs mitochondrial function, elevates reactive oxygen species (ROS) levels, and disrupts excitation–contraction coupling, collectively reducing neuromuscular transmission efficiency [80]. Together, these findings underscore the critical roles of chronic low-grade inflammation and oxidative stress in skeletal muscle aging and NMJ dysregulation, beyond the widely recognized contribution of motoneuron degeneration.

### 4.3. Impaired Immune Stem Cell Crosstalk

In rodent models, aging impairs the interplay between macrophages and satellite cells (SCs). Evidence from primary human ischemic muscle indicates that excessive M1 macrophage polarization disrupts SC self-renewal and promotes premature differentiation, thereby compromising regenerative capacity [81,82]. In contrast, the oxidative metabolism characteristic of M2-like macrophages is associated with increased anabolic signaling, reduced inflammation, and support of myogenic repair, indirectly maintaining a favorable neuromuscular microenvironment [83]. Because direct evidence in humans is limited, the role of these regulatory dynamics in the age-related decline in skeletal muscle regenerative capacity remains to be elucidated.

### 4.4. Autoimmune NMJ Disorders

Autoimmune myasthenia gravis (MG) is characterized by sporadic skeletal muscle weakness and increased serum concentrations of autoantibodies against components of the postsynaptic NMJ, such as AChR in 85% of MG cases, MuSK in 10% of cases, and less commonly LRP4 or argin [79]. However, experimental research in this field remains limited, mostly comprising observational human studies. In the future, it will be important to prioritize extensive and in-depth experimental studies to elucidate the action mechanisms of these antibodies, ultimately designing neutralizing antibodies or personalized therapeutic approaches [84,85].

Lambert–Eaton myasthenic syndrome (LEMS) is characterized by proximal lower limb weakness and elevated levels of pathogenic antibodies targeting voltage-gated calcium channels (VGCCs) on the presynaptic terminal [86]. While antibodies against VGCC-P/Q-type are well-established biomarkers in LEMS, recent studies revealed that VGCC-N-type antibodies show non-specific association with LEMS [87].

### 4.5. Genetic Mutations of NMJ Components

Congenital myasthenic syndrome (CMSs) is a group of genetic disorders resulting from mutations affecting multiple NMJ components. Since the first reports on CMS clinical cases in the 1970s, 35 CMS disease genes have been identified, affecting pre- or postsynaptic elements that impair NMJ function [88]. Clinical and pathologic features of CMS-relevant mutations were comprehensively reviewed in 2023 [89]. Notably, many pathogenic gene variants were initially identified in other diseases without significant NMJ deficiencies. Thus, future detailed clinical and electrophysiological examinations may identify further mutations that are relevant to NMJ function.

### 4.6. Biomarkers and Functional Correlations

C-terminal agrin fragment (CAF) reflects extracellular matrix (ECM) turnover within the synaptic cleft and increases with the progression of myopenia [61]. The upregulation of caveolin-3 (Cav3) expression may represent a compensatory response that enhances membrane curvature and stabilizes T-tubule structural integrity [62]. Electrophysiological measures, including jitter and motor unit potential complexity, can detect synaptic instability, whereas circulating neurofilament light chain (NfL) levels and NCAM^+^ fibers serve as indicators of concurrent axonal injury and regenerative activity [52,61]. Collectively, these findings support a model in which NMJ dysfunction acts as an early molecular trigger in the development of muscle decline, providing a framework for risk stratification before the development of overt muscular deterioration.

## 5. Conclusions and Future Perspectives

Elucidating the mechanisms of neuromuscular junction communication in health and disease is essential for the development of effective therapies for neuromuscular disorders. However, current understanding of human NMJ remains incomplete, largely because animal models exhibit heterogeneous synaptic morphologies and substantial age- or disease-related variability. Evidence from correlation studies indicates that large animal models may be more appropriate and accurate for understanding human NMJs and can lead to new insights into NMJ biology. Future advances will require systematic analyses of NMJs across diverse human muscles, developmental stages, and health conditions to clarify the mechanisms governing regeneration and neuronal remodeling. The evidence reviewed herein indicates that NMJ transmission instability can precede overt muscle atrophy, highlighting the need for longitudinal studies that directly link synaptic dysfunction to impaired recovery during aging. The development of new approaches that overcome the limitations of previous human studies will accelerate our understanding of NMJ repair and related diseases. The validation of electrophysiological and circulating biomarkers—including tremor characteristics, iMUNE, CAF, and NfL—may facilitate early risk stratification and the assessment of regenerative potential. Therapeutic strategies aimed at stabilizing agrin–LRP4–MuSK signaling or increasing reinnervation represent promising human-centered approaches for preserving NMJ connectivity and supporting functional recovery across the lifespan.

## Figures and Tables

**Table 1:** Experimental and Clinical Evidence Linking NMJ Structure and Function Across Species and Disease Contexts.

Species	Model	Target/Marker	Targeting Mechanism	Clinical Relevance/ Translation	Reference
human	clinical + ex vivo	DPAGT1 mutations	Impaired AChR glycosylation	Treatable CMS subtype	[58]
human	in vivo	CAF; NMJ genes; NF jitter	Mechanical unloading then resistance retraining	Disuse, rehab, denervation risk	[44]
human	cell lines (in vitro)	AChR α1β1δε	Cryo-EM with ACh/aBuTx; Fab35	Explains CMS variants’ gain/loss function	[38]
human	clinical + in vivo	CAF; MUP; NFM jiggle; segment jitter	Exercise preserves NMJ stability markers	Sarcopenia prevention strategy; aging MU remodeling; NMJ instability	[59]
human	clinical + in vivo	CAF; NfL; muscle architecture	Intramuscular EMG at 25% MVC	Sarcopenia prevention strategy	[60]
human	in vivo + ex vivo	CAF; NfL; iMUNE; NF jitter	Multimodal NMJ/MU assessment; no intervention	Early biomarkers for sarcopenia prevention	[61]
human	in vivo + ex vivo	Nogo-A; AChR; neurofilament	Correlative expression–denervation analysis	Nogo-A therapeutic target ALS	[48]
human	clinical + in vivo + ex vivo	CAF; AChR; SV2; NfL	Disuse-induced NMJ remodeling	Inactivity-related sarcopenia risk	[52]
human	ex vivo	Agrin LRP4 MuSK	Cryo-EM ternary complex assembly	Congenital myasthenia therapeutics	[62]
human, mouse	in vivo	AChR clusters; synaptic podosomes	Laminin-driven ECM signaling induces AChR clustering	Screening targets for neuromuscular disorders	[27]
human, mouse	ex vivo + cell-lines (in vitro)	(tSCs); S100, NG2; AChRs; SV2/2H3;	siRNA KD; EFNA5 overexpression	Aging NMJ loss; re-innervation support	[17]
human, mouse	ex vivo + cell-lines (in vitro)	CHRNE; EFNA5/SORBS2; GRIA2	Confocal morphometry comparison	Translational species differences;	[63]
human, mouse	ex vivo	AChR; SV2; NaV1.4	α-BTX; anti-SV2/NaV1.4	Human NMJ nanoscale organization	[40]
human, mouse	ex vivo	AChR; SV2/2H3; SNAP25;	Super-resolution imaging; proteomics	Species-specific NMJ translation	[18]
human, mouse	ex vivo	15-PGDH; PGE2;	Small-molecule PGDHi	Trauma; ALS; SMA; aging	[64]
human, mouse	in vivo + ex vivo	AMPK; PGC-1a; Dok7	Direct AMPK activation; muscle knockout	Aging; neuromuscular disorders	[65]
mouse, cat, dog, sheep, pig, human	ex vivo	SV2/2H3; α-BTX AChRs	NMJ-morph morphometry across species	Sheep best match the human NMJ	[19]
mouse, rat	in vivo	Kranocyte: cytoskeletal antibody (2166); CD34; CTB	Denervation-induced reactive expansion	ALS; Huntington’s muscle atrophy	[15]
mouse, rat	in vivo + ex vivo	Mitochondrial alarmins; PSC ERK	Axonal mitochondrial danger signaling	ALS-like dying-back mechanisms	[45]
mouse, rat	in vivo + ex vivo	NF200; α-bungarotoxin; CMAP	Delayed reinnervation after denervation	Nerve repair critical period	[66]
human; mouse; monkey	clinical + ex vivo	ColQ D-CTD; LRP4; AChR	Variant lowers ColQ–LRP4 binding	Explains COLQ CMS; informs therapy	[34]
mouse	in vivo + ex vivo	LRP4	Agrin-LRP4 signaling loss	CMS/MG mechanism	[28]
mouse	in vivo + ex vivo	Terminal Schwann cells; S100 + 7	SC ablation → postsynaptic defects first	NMJ degeneration; myasthenic relevance	[6]
mouse	in vivo + ex vivo	AChR; rapsyn; piccolo	AChRs edge-localized → AZ alignment	Efficient transmission; myasthenia context	[41]
mouse	in vivo + ex vivo	PGC1α; ERRα; Dok-7	Transcriptional promoter activation	Exercise-mediated NMJ restoration	[67]
mouse	in vivo + ex vivo	AChR BTX; synaptophysin	Mitochondrial transfer after toxin	NMJ repair after muscle injury	[68]
mouse	in vivo + ex vivo	P2X7; SV2/2H3; α-BTX	P2X7 agonist BzATP intraperitoneal dosing	Site-specific P2X7 modulation in ALS	[54]
mouse	in vivo + ex vivo	MLKL; MBP; PLP	S441 phosphorylation enables myelin breakdown	Nerve regeneration after injury	[53]
mouse	in vivo + ex vivo	Etv4; Pdzrn4; MuSK	AAV overexpression; muscle CRISPR	NMJ maintenance pathways	[51]
mouse	in vivo + ex vivo	mCherry-H2B myonuclei; BTX-labeled AChRs	HSA-Cre activates mCherry-H2B reporter	In vivo NMJ subsynaptic nuclei tracking	[50]
mouse	in vivo + ex vivo	Nav1.4; AnkR/B/G; α-BTX NMJ	Ankyrin loss blocks NMJ Nav1.4 clustering	NMJ excitability; prevents use-dependent failure	[42]
mouse	in vivo + ex vivo	IL-6/IL-6R; AChR-β; PGC1α; MEF2C	IL-6R signaling modulates AChR-β expression	Pre-sarcopenia NMJ target: IL-6	[69]
mouse	in vivo + ex vivo	SMN; U7 snRNP; Agrin; NMJ	AAV9 Lsm10/11 boosts U7 assembly	SMA NMJ rescue; adjunct therapy	[4]
mouse	in vivo + ex vivo	PIEZO1; Pax7 MuSCs	MuSC Piezo1 deletion; Rho rescue	Muscle repair; sarcopenia relevance	[10]
mouse	in vivo + ex vivo	YAP1, TAZ, TEAD1/4; synaptic genes	Muscle Cre knockout; CRISPR; AGRN media	Mechanisms for NMJ weakness disorders	[9]
mouse	ex vivo	SV2 2H3; α-BTX AChR	NMJ-morph quantification workflow	Benchmark for NMJ studies	[70]
mouse	ex vivo	P2Y1; TPSCs; GCaMP3	Purinergic Ca^2+^ signaling blockade	Muscle fatigue modulation	[46]
mouse	ex vivo	Muscle nAChR; GsMTx-4	Lipid-partitioning toxin; fast desensitization	Channel desensitization; therapeutic insight	[36]
mouse	ex vivo	MuSK; Lrp4; AChRα/β	Agrin increases MuSK endocytosis	MuSK trafficking; NMJ maintenance	[29]
mouse	ex vivo	CTGF/CCN2, LRP4, MuSK	CTGF enhances LRP4–MuSK signaling	Congenital myasthenic mechanisms	[20]
mouse	in vivo	Collagen XIII; AChRs	Col13a1 loss → presynaptic regeneration failure	CMS19; nerve injury recovery	[30]
mouse	in vivo	MuSK, anti-MuSK IgG, Treg	Oral MuSK → Treg-mediated immune suppression	MuSK-MG antigen-specific therapy	[5]
mouse	in vivo	nAChR; Cx43/45 hemichannels	ACh agonists; pyridostigmine; Cx43/45 KO	Targets for atrophy; reinnervation therapies	[37]
mouse	in vivo	Vegf-A; CD68 macrophages; tSC S100	LysMCre Vegf-A cKO; CBZ VegfR2	Targets to improve motor reinnervation	[7]
mouse	in vivo	GD1b; complement MAC; pSC S100B	Anti-GD1b antibody plus human complement	GBS distal axon debris clearance	[8]
mouse	in vivo	MuSK agonist antibody X-17	MuSK activation independent of Agrin	ALS NMJ protection; lifespan extension	[71]
lamprey	ex vivo	CaV2.1/2.2/2.3; presynaptic AZ	Cell-attached patch; LLSM Ca^2+^ imaging	Synaptic dysfunction mechanism insight	[25]
xenopus, mouse	in vivo + ex vivo	Caveolin-3; lipid rafts; AChR	Cav3 MO/shRNA; MβCD cholesterol depletion	Junctional folds in muscular dystrophy	[43]
mouse, dog	in vivo	Muscle fibers; AChRs; SCs	Fiber degeneration/regeneration → NMJ remodeling	DMD progression; therapeutic timing	[72]
mouse, frog	in vivo	AChE; basal lamina	BL-anchored AChE → efficient ACh hydrolysis	Myasthenia; synaptic transmission fidelity	[26]
rat	in vivo	Mast cells; tryptase+; c-Kit+	Mast degranulation → NMJ denervation	ALS progression; anti-inflammatory target	[16]

## Data Availability

No new data were created or analyzed in this study. Data sharing is not applicable to this article.

## Abbreviations

The following abbreviations are used in this manuscript:

ACh	Acetylcholine
AChE	Acetylcholinesterase
AChR	Acetylcholine Receptor
Akt	Protein Kinase B (AKT)
ALS	Amyotrophic Lateral Sclerosis
AMPK	AMP-Activated Protein Kinase
ATP	Adenosine Triphosphate
AZ	Active Zone
CAF	C-terminal Agrin Fragment
Cav3	Caveolin-3
CCN2/CTGF	Cellular Communication Network Factor 2/Connective Tissue Growth Factor
ChAT	Choline Acetyltransferase
CMAP	Compound Muscle Action Potential
COLQ	Collagen Q (AChE-anchoring collagen)
cryo-EM	Cryo-Electron Microscopy
Dok-7	Downstream of Tyrosine Kinase 7
ECM	Extracellular Matrix
EPP	Endplate Potential
EMG	Electromyography
FAK	Focal Adhesion Kinase
iMUNE	Motor Unit Number Estimation (incremental MUNE)
IL-6	Interleukin-6
iPSC	Induced Pluripotent Stem Cell
LRP4	Low-Density Lipoprotein Receptor-Related Protein 4
MAPK	Mitogen-Activated Protein Kinase
MuSK	Muscle-Specific Kinase
Munc13/Munc18	Mammalian Unc-13/Unc-18 homologs
nAChR	Nicotinic Acetylcholine Receptor
Nav1.4	Skeletal-Muscle Voltage-Gated Sodium Channel α-subunit
NCAM	Neural Cell Adhesion Molecule
NfL	Neurofilament Light Chain
NMJ	Neuromuscular Junction
PI3K	Phosphoinositide 3-Kinase
PSC	Perisynaptic Schwann Cell
PGC-1α	Peroxisome Proliferator-Activated Receptor Gamma Coactivator-1α
RIM	Rab3-Interacting Molecule
ROS	Reactive Oxygen Species
SC	Satellite Cell
SNARE	Soluble NSF Attachment Protein Receptor
SV2	Synaptic Vesicle Glycoprotein 2
TAZ	Transcriptional Co-Activator with PDZ-binding motif
TNF-α	Tumor Necrosis Factor-α
tSC	Terminal Schwann Cell
VGCC	Voltage-Gated Calcium Channel
YAP	Yes-Associated Protein
MG	Myasthenia gravis
SMA	Spinal muscular atrophy
